# Retraction Note: Bioprocessing of camptothecin from *Alternaria brassicicola*, an endophyte of *Catharanthus roseus*, with a strong antiproliferative activity and inhibition to Topoisomerases

**DOI:** 10.1186/s12934-026-03171-y

**Published:** 2026-11-09

**Authors:** Nouran A. A. Abd El-Hady, Abdelaleim I. ElSayed, Khalid M. Wadan, Sayed S. El-Saadany, Ashraf S. A. El‑Sayed

**Affiliations:** 1 Enzymology and Fungal Biotechnology Lab, Botany and Microbiology Department, Faculty of Science, Zagazig University, Zagazig, 44519 Egypt; 2 Biochemistry Department, Faculty of Agriculture, Zagazig University, Zagazig, 44519 Egypt

**Retraction Note: Microbial Cell Factories (2024) 23:214**

**https://doi.org/10.1186/s12934-024-02471-5**

The Editor-in-Chief has retracted this article. After publication, concerns were raised regarding the data presented in the figures. Specifically, the article appears to share a large amount of control data with the authors’ earlier article [1] without appropriate atttribution. Additionally, several TLC blots appear highly similar between Fig. 6A and B.

The authors have explained that the experiments presented in the two studies indeed shared the same control groups and stated that Fig. 6 contained an inadvertent error. However, they have not provided the associated raw data upon request. The Editor-in-Chief therefore no longer has confidence in the presented data.

Nouran A. A. Abd El-Hady and Ashraf S. A. El-Sayed do not agree with this retraction. Abdelaleim I. ElSayed, Khalid M. Wadan and Sayed S. El-Saadany have not responded to any correspondence from the editor or publisher about this retraction.
